# Sleeve gastrectomy with antral resection provides more effective weight loss in patients with super obesity

**DOI:** 10.1007/s00423-025-03607-w

**Published:** 2025-01-11

**Authors:** Ahmet Tarik Hamantepe, Emre Gönüllü, Gizem Fırtına, Onur İlhan, Adem Yüksel, Kerem Karaman

**Affiliations:** 1 Faculty of Medicine Training and Research Hospital, Department of Gastrointestinal Surgery, Sakarya University, Sakarya, Turkey; 2 Department of Gastrointestinal Surgery, Kocaeli State Hospital, Kocaeli, Turkey

**Keywords:** Super obesity, Sleeve gastrectomy with antrectomy, Postoperative nausea, Vomiting

## Abstract

**Background:**

Obesity is a growing health issue that contributes to numerous diseases and lowers quality of life. In patients with super obesity (BMI > 50 kg/m²), bariatric surgery, particularly laparoscopic sleeve gastrectomy (LSG), is a common treatment option. However, the role of antral resection (AR) in LSG remains unclear, especially in this high-risk population. This study aims to compare the effectiveness of LSG with antral resection (LSG-AR) and LSG with antrum preservation (LSG-AP) on weight loss and postoperative complaints in patients with super obesity.

**Methods:**

The medical records of patients with a BMI > 50 kg/m² who underwent LSG between 2016 and 2022 were retrospectively reviewed. Weight data were collected at admission, and at the first, second, and fifth-year follow-up. Patients were divided into two groups based on LSG-AR or LSG-AP procedures.

**Results:**

Seventy-two patients were included. No significant difference was found in postoperative vomiting complaints between the groups (*p* = 0.67). First-year outcomes showed no significant differences in weight, BMI, or % Total Weight Loss (%TWL). However, second and fifth-year %TWL values were significantly higher in the LSG-AR group (*p* = 0.003 for both).

**Conclusion:**

LSG-AR provides more effective long-term weight loss in patients with super obesity. Early postoperative vomiting complaints diminish over time, suggesting LSG-AR as a viable one-stage procedure for this patient population.

## Highlights

Sleeve gastrectomy with antral resection achieves effective weight loss.

Antral resection causes more nausea and vomiting in the early period.

Nausea and vomiting seen after antral resection decrease reasonably over time.

Sleeve gastrectomy with antral resection can be preferred in patients with super obesity.

## Introduction

Obesity is a health problem whose prevalence increases day by day and reduces the quality of life. Moreover, it is involved in the etiology of many diseases [1, 2]. Exercise and changing eating habits are the first steps in the treatment of obesity [3–5]. Pharmacological treatments and bariatric surgeries come to the fore in patients with obesity when adequate weight loss cannot be achieved with these lifestyle changes [6].

The fact that LSG is seemingly easy and the learning curve is shorter than other bariatric surgical procedures makes it more commonly performed among bariatric surgical procedures [7]. However, LSG is not a standardized method. There is no consensus among surgeons about the diameter of the boogie to be used in LSG, whether reinforcing the stapler line, or whether an antrectomy should be performed [8–10].

Body mass index > 40 is defined as Class III obesity, and BMI > 50 kg/m^2^ is defined as super obesity [11, 12]. Numerous studies have been evaluated the effect of preservation of the antrum in LSG on postoperative complaints and weight loss in Class III patients with obesity [13–18]. However, there are no studies in the literature evaluating the effects of antrectomy in the super patient with obesity group, where sleeve gastrectomy is more complicated and perioperative morbidity-mortality is higher than in patients with severe obesity [19].

The aim of the present study is to compare in patients with super obesity LSG-AR with LSG-AP on weight loss and postoperative patient complaints.

## Materials and methods

This study was conducted following the approval of the University Ethics Committee (E-16214662-050.01.04-319690-198). The medical records of patients with a BMI > 50 kg/m^2^ who underwent LSG between 2016 and 2022 were retrospectively reviewed. Information on age, gender, comorbid diseases, surgery date, early postoperative complications, presence of vomiting-reflux complaints in the postoperative period (if any, duration), weight at admission, and first-year and second-year, and fifth-year follow-up examinations were extracted from the patient files. Vomiting lasting more than four weeks was considered as long-term vomiting [20]. Early postoperative complications were recorded by calculating Clavien Dindo’s scoring.

We contacted the patients who completed the fifth postoperative year by phone, measured their weight, and recorded them. Charlson Comorbidity Index (CCI) and BMI at admission, as well as BMI and Total weight loss percent (%TWL) at follow-up examinations, were calculated and recorded [21, 22].

### Patient enrollment

#### Inclusion criteria

Patients who agreed to participate in the study, whose file information was complete and organized, BMI > 50 kg/m^2^, between 18 and 65 years of age, who had not undergone previous bariatric surgery, and who had no gastroesophageal reflux detected on preoperative endoscopy.

#### Exclusion criteria

Patients who refused to participate in the study, whose file information was incomplete or irregular, whose BMI was ≤ 50 kg/m^2^, whose age was < 18 years or > 65 years, who had undergone previous bariatric surgery, and whose preoperative endoscopy revealed gastroesophageal reflux.

All patients were operated on by a single surgeon. While antrum-preserving surgery was performed for all patients before February 2018, antrectomy was performed after February 2018.

**Fig. 1:**
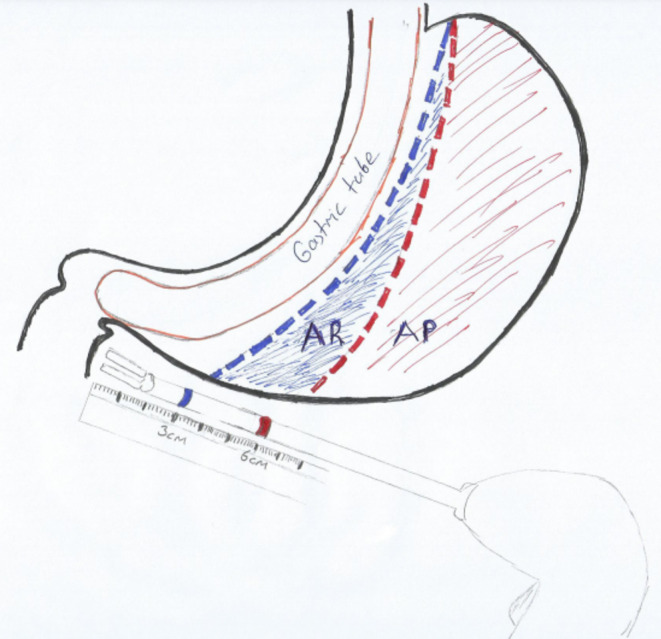
Calculating the distance from the pylorus to begin gastric transection with a stapler

The distinction between AR/AP was made as follows: The sealing device was marked by staining with a sterile pen at 3 and 6 cm from the tip (Fig. 1). Calculation of gastric division according to AR or AP was made after the tip of the sealing device was fixed to the pylorus. Gastric division was started from 3 cm to the pylorus in AR patients and from 6 cm in AP patients.

Patients were divided into two groups as patients who underwent antrectomy during the LSG procedure (LSG-AR) (first group) and patients who did not undergo antrectomy (LSG-AP) (second group). Statistical analysis was performed between the two groups in terms of the variables mentioned above.

### Surgical procedure

LCD was performed in the French position. We placed a 36 F orogastric bougie into the stomach prior to surgery. The greater curvature was transected starting 3 cm away from pylorus in LSG-AR, and 6 cm away from the pylorus in (LSG-AP).

### Postoperative follow-up

We performed follow-up examinations at the first, 3rd, 6th, 12th, 18th and 24th months. Thereafter, annual follow-up. Blood parameters and patients’ weights were recorded. Patients who completed the fifth postoperative year were called for follow-up by phone, and examination datas were recorded.

### Statistical analysis

We performed descriptive analyses on the study population. The Kolmogorov-Smirnov test was used to evaluate whether numerical variables were distributed normally. The independent sample t-test for parametric numerical variables and the Mann-Whitney U test for non-parametric numerical variables were performed. Categorical variables were analyzed with the Chi-Square test. Categorical variables were presented as a count and percentage. The non-parametric numeric variables were presented as median (minimum-maximum). Parametric numeric variables were presented as mean ± standard deviation. A p-value < 0.05 was considered significant. Statistical analysis was performed using SPSS statistical software (IBM SPSS Statistics, Version 23.0. Armonk, NY: IBM Corp.).

## Results

**Fig. 2:**
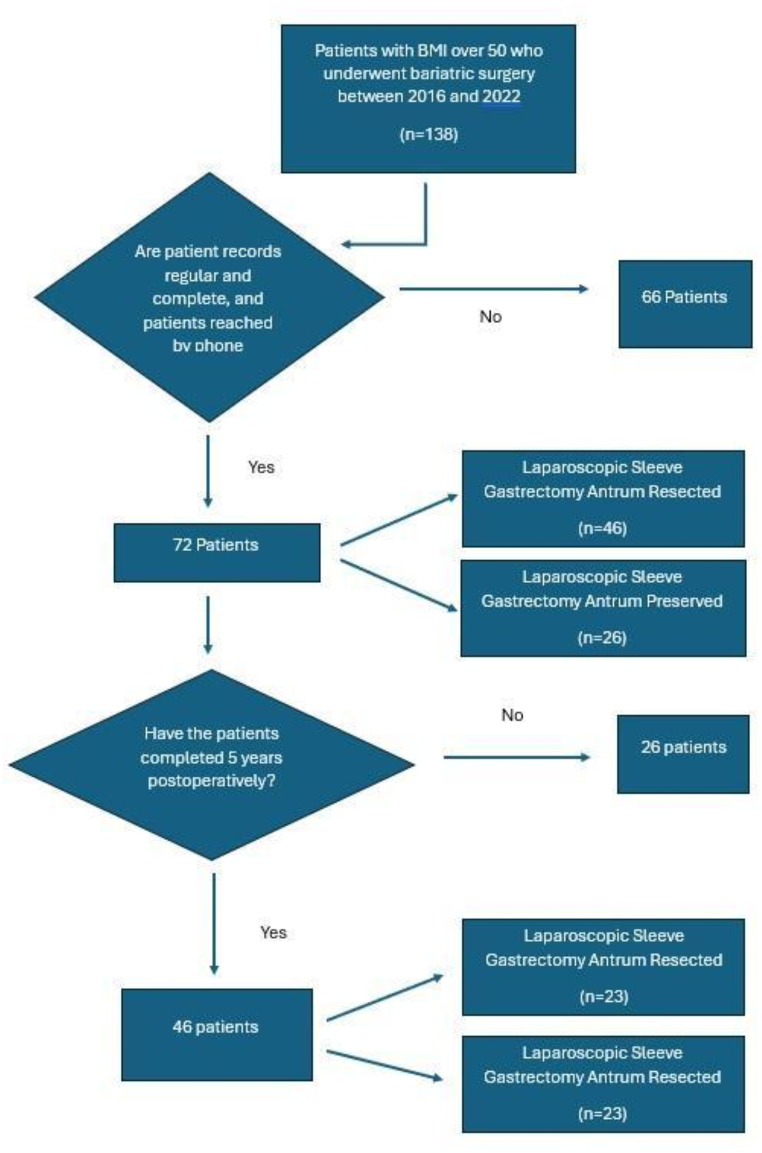
Flowchart of study design

During the study period, 138 patients with a BMI > 50 kg/m^2^ underwent bariatric surgery. Of these patients, 66 were excluded from the study because their records were incomplete or irregular or could not be reached by phone. Seventy-two patients with complete and organized records and who were contacted by phone were included in the study. Forty-six (63.9%) patients who underwent the LSG-AR procedure were included in the first group, and 26 (36.1%) patients who underwent the LSG-AP procedure were included in the second group (Fig. 2) (Table 1). First and second-year follow-up data on all patients was available in the files.

**Table 1:** Patient characteristics, demographic, and perioperative variables

		LSG-AR (*n* = 46)	LSG-AP (*n* = 26)	Total (*n* = 72)	*p* value
Age		36.26 ± 11.636	39.73 ± 13.939	37.51 ± 12.532	0.262*
Gender	Female	37 (64.9%)	20 (35.1%)	57 (79.1%)	0.768**
	Male	9 (60%)	6 (40%)	15 (20.9%)	
Height		163.67 ± 9.019	162.96 ± 8.628	163.42 ± 8.825	0.0745*
Weight		140.5 (100–270)	144 (122–174)	142 (100–270)	0.467***
Body Mass Index		52.826 (50-78.8)	53.1 (50.1–62.5)	53 (50-78.8)	0.982***
Charlson Comorbidity Index		0 (0–1)	0 (0–3)	0 (0–3)	0.292***
Clavien Dindo Score		1 (1–3)	1 (1–3)	1 (1–3)	0.507***
Duration of Hospitalization		4 (4–14)	4 (4–7)	4 (4–14)	0.340***

LSG-AR: Laparoscopic Sleeve Gastrectomy-Antrum Resected

LSG-AP: Laparoscopic Sleeve Gastrectomy-Antrum Preserved

* The Independent Samples T-test, ** The Chi-Square test, ***The Mann Whitney-U test

Forty-six (63.9%) patients completed the fifth postoperative year. Among these patients, 23 (50%) patients underwent LSG-AR, and 23 (50%) patients underwent LSG-AP (Fig. 2).

The mean age of the patients was 37.51 ± 12.532 years. The difference between the groups regarding mean age was not statistically significant (*p* = 0.262) (Table 1). Fifty-seven (79.1%) of the patients were female and 15 (20.9%) were male. The difference between the groups regarding gender distribution was not statistically significant (*p* = 0.768) (Table 1).

The mean height, median weight, and BMI of the patients were 163.42 ± 8.825 cm, 142 kg (100–270), and 53 kg/m^2^ (50-78.8), respectively. Differences between the groups regarding height, weight, or BMI were not statistically significant (*p* = 0.0745, 0.467, 0.982, respectively) (Table 1).

The differences between the two groups regarding comorbidities and early postoperative complications were not statistically significant (*p* = 0.292, *p* = 0.507, respectively) (Table 1). Cardiac problems were observed in 5 patients (arrhythmia in 4 patients, resistant hypertension in 1). Red blood cell suspension and/or fresh frozen plasma replacement was required in 6 patients due to bleeding. Pulmonary complications were observed in 6 patients (pneumonia in 2 patients, atelectasis in 2 patients, asthma attack in 2 patients). 1 patient was reoperated on the 1st postoperative day due to leak. This patient was discharged on the 6th postoperative day.

The information on whether the patients complained of vomiting in the early postoperative period was not recorded in two patient files; 49 (70%) of 70 patients did not complain of vomiting, and 21 (30%) complained of vomiting. Vomiting was more common in the LSG-AR group (18 (40%) vs. 3 (12%)). The difference between the two groups regarding vomiting was statistically significant (*p* = 0.016). We found that vomiting persisted longer in the LSG-AR group than in the LSG-AP group (0 weeks (0–71 weeks) vs. 0 weeks (0–13 weeks)). This difference between the two groups was statistically significant (*p* = 0.01). However, no statistically significant difference was found when the groups were compared regarding complaints of vomiting lasting longer than four weeks (*p* = 0.67) (Table 2).

**Table 2:** Vomiting and reflux complaints in the early postoperative period

		LSG-AR (*n* = 46)	LSG-AP (*n* = 26)	Total	*p*-value
Vomiting	No	27 (60%)	22 (88%)	49 (70%)	0.016*
	Yes	18 (40%)	3 (12%)	21 (30%)	
Duration of the vomiting complaint (weeks)		0 (0–71)	0 (0–13)	0 (0–71)	0.01**
Vomiting lasting more than four weeks	No	33 (71.7%)	24 (92.3%)	57 (79.2%)	0.67**
	Yes	13 (28.3%)	2 (7.7%)	15 (20.8%)	
Reflux	No	33 (75%)	20 (80%)	53 (76.8%)	0.770*
	Yes	11 (25%)	5 (20%)	16 (23.2%)	

LSG-AR: Laparoscopic Sleeve Gastrectomy-Antrum Resected

LSG-AP: Laparoscopic Sleeve Gastrectomy-Antrum Preserved

* The Chi-Square Test, **The Mann Whitney U test

While the information on whether the patients had gastro-esophageal reflux complaints in the early postoperative period was not recorded in three patient files, it was found that 53 (76.8%) of 69 patients did not have reflux complaints, and 16 (23.2%) had reflux complaints. Although reflux complaints were more common in the LSG-AR group (11 (25%) vs. 5 (20%)), the difference between the groups was not statistically significant (*p* = 0.770) (Table 2).

**Fig. 3:**
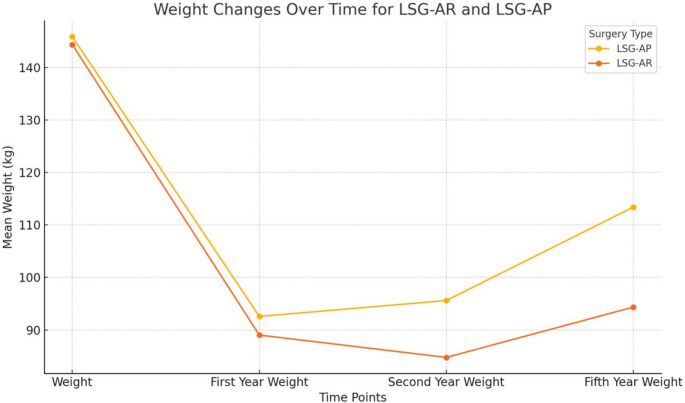
Weight change of LSG-AP and LSG-AR over the years. LSG-AR: laparoscopic sleeve gastrectomy-antrum Resected, LSG-AP: laparoscopic sleeve gastrectomy-antrum preserved

Weight changes of the groups over the years are given in Fig. 3.

### Evaluation of weight loss in the first postoperative year

The median postoperative first-year control weight was 85 kg (60–180) in the LSG-AR group and 90 kg (57–127) in the LSG-AP group. The mean first-year control BMI values were 33.107 ± 5.117 kg/m^2^ in the LSG-AR group and 34.931 ± 5.324 kg/m^2^ in the LSG-AP group. The mean first-year control %TWL value was 38.337 ± 7.639 in the LSG-AR group and 36.223 ± 9.627 in the LSG-AP group.

Differences in weight, BMI, and first-year control %TWL values between the groups were not statistically significant (*p* = 0.0687, *p* = 0.157, *p* = 0.309, respectively) (Table 3).

**Table 3:** Postoperative weight loss analysis

	LSG-AR (*n* = 46)	LSG-AP (*n* = 26)	*p*-value
First-year control weight	85 (60–180)	90 (57–127)	0.0687*
Second-year control weight	84.35 ± 13.40	95.38 ± 15.600	0.002**
Fifth-year control weight	90 (67–145)	107 (79–180)	0.005*
First-year control BMI	33.107 ± 5.117	34.931 ± 5.324	0.157**
Second-year control BMI	31.473 ± 4.301	35.976 ± 5.527	0.000**
Fifth-year control BMI	35.437 ± 7.108	42.410 ± 8.237	0.004**
First-year control %TWL	38.337 ± 7.639	36.223 ± 9.627	0.309**
Second-year control %TWL	41.111 ± 8.455	34.266 ± 10.135	0.003**
Fifth-year control %TWL	33.519 ± 14.285	22.936 ± 15.556	0.021**

LSG-AR: Laparoscopic Sleeve Gastrectomy-Antrum Resected

LSG-AP: Laparoscopic Sleeve Gastrectomy-Antrum Preserved

* The Mann Whitney U test, ** The Independent samples T-test

### Evaluation of weight loss in the second postoperative year

The patients’ mean weight in the second postoperative year was 84.35 kg ± 13.40 in the LSG-AR group and 95.38 kg ± 15.60 in the LSG-AP group. The patients’ mean weight in the LSG-AR group was significantly lower (*p* = 0.002) (Table 3).

The patients’ mean second-year control BMI values were 31.473 ± 4.301 kg/m^2^ in the LSG-AR group and 35.976 ± 5.527 kg/m^2^ in the LSG-AP group. The mean second-year control BMI values of the patients in the LSG-AR group were significantly lower (*p* = 0.000) (Table 3).

The mean second-year control %TWL values were 41.111 ± 8.455 in the LSG-AR group and 34.266 ± 10.135 in the LSG-AP group. The mean second-year %TWL values of the patients in the LSG-AR group were significantly higher (*p* = 0.003) (Table 3).

### Evaluation of weight loss in the fifth postoperative year

The median weight of the patients in the fifth postoperative year was 90 kg (67–145) in the LSG-AR group and 107 kg (79–180) in the LSG-AP group. The median weight of the patients in the LSG-AR group was significantly lower (*p* = 0.005) (Table 3).

The mean BMI values at the fifth-year follow-up were 35.437 ± 7.108 kg/m^2^ in the LSG-AR group and 42.410 ± 8.237 kg/m^2^ in the LSG-AP group. The mean second-year control BMI values of the patients in the LSG-AR group were significantly lower (*p* = 0.004) (Table 3).

The mean fifth-year control %TWL values were 33.519 ± 14.285 in the LSG-AR group and 22.936 ± 15.556 in the LSG-AP group. The mean fifth-year %TWL values of the patients in the LSG-AR group were significantly higher (*p* = 0.003) (Table 3).

## Discussion

Although studies show that performing an antrectomy in LSG causes an increase in postoperative vomiting complaints. Contrary, there are studies showing that performing an antrectomy does not statistically significantly increase vomiting and reflux complaints [15, 16, 23]. Robert M. et al. [15] propose that vomiting complaints are higher in the early postoperative period in patients with small remnant stomachs left after sleeve gastrectomy, but this difference disappears in the long term. Diab ARF et al. [9] obtained results supporting this finding in their study conducted in 2023 with groups of patients with and without antrectomy. In the present study, consistent with the literature, we observed that vomiting occurred more frequently and lasted longer in the early postoperative period in patients who underwent antrectomy, but this difference disappeared in the long term, and moreover, antrectomy had no effect on reflux complaints.

It is a concern among some bariatric surgeons that performing an antrectomy in the stomach, whose volume has been reduced by sleeve gastrectomy, will further reduce the gastric volume and lead to an increase in intraluminal pressure, which may jeopardize stapler safety [24–26]. However, many authors suggest that LSG-AR poses no additional risk to stapler safety compared to LSG-AP [27–29]. In our study, we found no significant statistical difference between the LSG-AR and LSG-AP groups regarding early complications, similar to studies showing that both methods were equally safe.

There are many studies comparing LSG-AR and LSG-AP regarding effective weight loss, and contradictory results have been obtained [13, 30]. However, to the best of our knowledge, we have not found any other study comparing the super-obese in terms of LSG-AR and LSG-AP. ElGeidie A et al. [28] suggest that LSG-AR provides more effective weight loss in the first six months, but both methods have similar success rates in terms of weight loss in the first postoperative year. Maklad A et al. [14] also found that the success rate of the two methods was similar in terms of weight loss at the end of the 6th month. Jacobs M et al. [31] suggest no statistically significant difference exists between the %TWL rates of patients who underwent LSG-AR and LSG-AP in the first and second year. Eskandaros MS stated in his study that the %TWL rate was statistically significantly higher in patients who underwent LSG-AR at the 6th and 12th-month controls [18]. In this study conducted with patients with super obesity, the difference between the two groups regarding %TWL rates in the first year follow-up was not statistically significant. However, we observed more effective weight loss in the LSG-AR group in the second and fifth-year controls. There is no consensus among authors on how far the transection should start from the pylorus when performing anrectomy in sleeve gastrectomy operations. Some authors define starting gastric transection one centimeter from the pylorus as LSG-AR, while some authors define starting resection four centimeters from the pylorus as LSG-AR [9]. The fact that inadequate antrum resection was performed due to gastric transection beginning from a long distance to the pylorus in the studies may have led to the finding that LSG-AR and LSG-AP were equally effective in terms of weight loss. The findings of the study by Avlanmış O et al. [32] support this hypothesis. Although we did not measure the remaining gastric volume after gastric resection in our study, the fact that we routinely performed gastric transection starting three centimeters from the pylorus and performed adequate antrum resection may have provided more effective weight loss in the LSG-AR group.

The antrum performs reverse peristalsis. Therefore, while gastric reserve decreases with antrectomy, pyloric dysfunction occurs at the same time. Therefore, alkaline reflux increases. This causes nausea and vomiting. Therefore, we think that for reducing reflux symptoms, the patient takes less oral food and loses more weight.

The multi-stage or stepwise therapeutic approach is being suggested by some authors in patients with super obesity [33, 34]. However, this strategy itself may cause morbidities, may not significantly reduce perioperative complications, and may reduce effective weight loss [35, 36]. Considering there is no consensus among authors regarding the multi-stage or stepwise therapeutic approach, the LSG-AR method, which provides effective weight loss in the long term, may be preferred instead of staged treatment in patients with super obesity.

### Limitations of the study

Our study has some limitations. It is a retrospective study and single-center. In addition, although we are a high-volume clinic, a limited number of patients were included in the study due to missing data. Considering that there were mobility restrictions and changes in people’s eating habits during the COVID-19 pandemic, the time interval in which the study was conducted, including the COVID-19 pandemic process, may have affected the results.

## Conclusion

In the case of patients with super obesity, LSG-AR has no superiority in weight loss in the first year compared to LSG-AP. Further, it causes more complaints of nausea and vomiting in the early postoperative period. However, in the long term, LSG-AR does not cause more vomiting than LSG-AP and may provide weight loss much more effectively. Moreover, the success of LSG-AR in achieving permanent and effective weight loss in the long term suggests that it can be used as a one-stage procedure in patients with super obesity.

## Data Availability

No datasets were generated or analysed during the current study.
